# Comparative efficacy of second-generation androgen receptor inhibitors for treating prostate cancer: A systematic review and network meta-analysis

**DOI:** 10.3389/fendo.2023.1134719

**Published:** 2023-03-09

**Authors:** Xiangyu Chen, Qihua Wang, Yang Pan, Shangren Wang, Yuezheng Li, Hao Zhang, Mingming Xu, Hang Zhou, Xiaoqiang Liu

**Affiliations:** Department of Urology, Tianjin Medical University General Hospital, Tianjin, China

**Keywords:** second-generation androgen receptor inhibitors, metastatic hormone-sensitive prostate cancer, non-metastatic castration-resistant prostate cancer, metastatic castration-resistant prostate cancer, enzalutamide, apalutamide, darolutamide

## Abstract

**Introduction:**

**S**econd-generation androgen receptor inhibitors (SGARIs), namely enzalutamide, apalutamide, and darolutamide, are good for improving survival outcomes in prostate cancer patients, but some researchers have shown that using SGARIs increases side effects, which complicates clinicians’ choice of. Therefore, we performed this network meta-analysis to assess the efficacy and toxicity of several SGARIs in the treatment of patients with metastatic hormone-sensitive prostate cancer (mHSPC), non-metastatic castration-resistant prostate cancer (nmCRPC), and metastatic castration-resistant prostate cancer (mCRPC).

**Methods:**

We searched PubMed, EMBASE and Cochrane Library databases from January 2000 to December 2022 to identify randomized controlled studies associated with SGARIs. We use Stata 16.0 and R 4.4.2 for data analysis, hazard ratio (HR) with 95% confidence intervals (CI) were used to assess the results.

**Results:**

This meta-analysis included 7 studies with a total of 9488 patients. In mHSPC, enzalutamide and darolutamide had a positive effect on overall survival (OS) (HR, 0.70; 95% CI, 0.59-0.82), but we did not find a difference in their efficacy to improve OS (HR, 1.19; 95% CI, 0.75-1.89). Also in nmCRPC, enzalutamide, apalutamide and darolutamide were beneficial for metastasis-free survival (MFS) (HR, 0.32; 95% CI, 0.25-0.41). Compared to darolutamide, enzalutamide (HR, 0.71; 95% CI, 0.54-0.93) and apalutamide (HR, 0.68; 95% CI, 0.51-0.91) prolonged MFS, but there was no difference in efficacy between enzalutamide and apalutamide (HR, 0.97; 95% CI, 0.73-1.28). Finally in mCRPC, there was no significant difference in indirect effects on OS between pre- and post-chemotherapy enzalutamide (HR, 0.89; 95% CI, 0.70-1.13). However, using enzalutamide before chemotherapy to improve radiographic progression-free survival (rPFS) was a better option (HR, 2.11; 95% CI, 1.62-2.73).

**Conclusion:**

The SGARIs used in each trial were beneficial for the primary endpoint in the study. Firstly there was no significant difference in the effect of enzalutamide and darolutamide in improving OS in patients with mHSPC. Secondly improving MFS in patients with nmCRPC was best achieved with enzalutamide and apalutamide. In addition both pre- and post-chemotherapy use of enzalutamide was beneficial for OS in mCRPC patients, but for improving rPFS pre-chemotherapy use of enzalutamide should be preferred.

The INPLASY registration number of this systematic review is INPLASY202310084.

## Introduction

Prostate cancer is the second most common cancer worldwide and the sixth leading cause of cancer death in men. Age, African ancestry, and family history are the only established risk factors of prostate cancer (1). Prostate cancer develops when the binding of androgens to the androgen receptor (AR) triggers a key specific oncogenic transcriptional program (2). Androgen deprivation therapy (ADT) inhibits AR activation and/or the synthesis and release of pituitary gonadotropins. This could be accomplished through the use of gonadotropin hormone-releasing hormone (GnRH) agonists with or without androgen receptor inhibitors (ARIs), GnRH antagonists, or bilateral orchiectomy (3).

Approximately 80-90% of prostate cancers are androgen-dependent at initial diagnosis, and 27-53% of early-stage prostate cancers progress with biochemical recurrence. Although ADT and/or the use of first-generation competitive androgen receptor inhibitors can temporarily arrest tumor growth, most men develop castration-resistant prostate cancer (CRPC) after 14-20 months on ADT therapy (4). Additionally, 3–5% of patients present with metastatic disease either before or after a cure attempt, which is known as metastatic hormone-sensitive prostate cancer (mHSPC) (5, 6). ARI are widely used in clinical practice, and the use of ARIs with ADT, rather than ADT alone, has been postulated to improve patient outcomes (7). Yet the practice of adding first-generation ARIs such as bicalutamide to ADT in the setting of castration resistance is based on single-arm studies in a modest number of men that suggested limited benefit lasting no more than 3 to 6 months (8, 9). In the past few years, the US Food and Drug Administration has approved three second-generation androgen receptor inhibitors (SGARIs), namely enzalutamide, apalutamide, and darolutamide, to treat prostate cancer. Compared to first-generation ARIs, SGARIs possess a higher affinity for AR and improve survival outcomes (10). A STRIVE trial found that enzalutamide significantly reduced the risk of disease progression or death by 76% compared with bicalutamide, and the benefit of enzalutamide over bicalutamide was robust, with a more than 1 year prolongation of median radiographic progression-free survival (rPFS) (11). However, the potential side effects of SGARIs and patient resistance cannot be ignored (12). A meta-analysis has indicated that the administration of SGARIs is associated with a significantly increased risk of cardiovascular events including stroke, heart failure, and peripheral vascular disease (13). The overall benefits of different types of SGARIs are difficult to assess given the need to weigh the risk of side effects against the efficacy of SGARIs for treating prostate cancer. Therefore, we performed an indirect comparison and network meta-analysis of several SGARIs to assess their efficacy and toxicity in the treatment of patients with mHSPC, non-metastatic CRPC (nmCRPC), and metastatic CRPC (mCRPC), which should in turn inform ARI selection for more effective treatments (14).

## Methods

The INPLASY registration number of this systematic review is INPLASY202310084. This study included randomized controlled studies. Participants were patients with mHSPC,nmCRPC or mCRPC, and the efficacy and toxicity of SGARIs was further demonstrated by comparing the primary and secondary endpoints of patients with enzalutamide intervention to those with placebo intervention.

### Study selection

**Figure 1:**
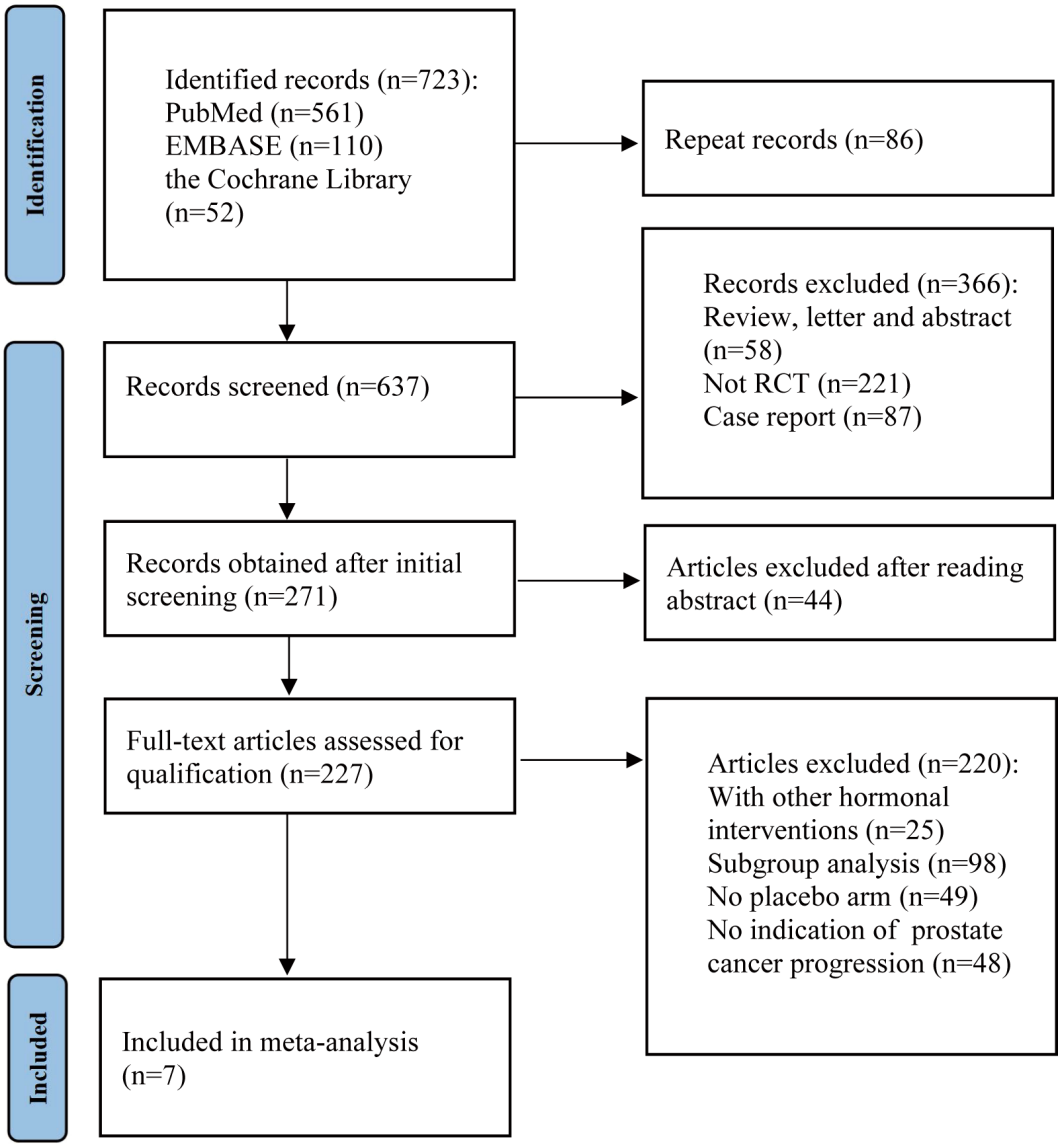
Flow chart for PRISMA-based articles screening.

The PRISMA guidelines were followed as this systematic review and meta-analysis was being conducted (15). As detailed in the PRISMA flow chart (**Figure 1**), we queried the databases PubMed, EMBASE, and the Cochrane Library with text and keywords, both as MeSH (Medical Subject Headings) terms and text words, to find studies published between January 2000 to December 2022. Search terms used in the search strategy: “prostate cancer,” “androgen receptor,” “endocrine therapy,” “darolutamide,” “enzalutamide,” “apalutamide,” “metastatic hormone-sensitive prostate cancer,” “nonmetastatic castration-resistant prostate cancer,” and “metastatic castration-resistant prostate cancer.” For studies in different journals that have overlapping data, duplicated data, or the same authors, we used the most recent or most comprehensive study. Review articles, letters, commentaries, case reports, and preclinical studies were excluded.

### Inclusion and exclusion criteria

The inclusion criteria for eligible studies were as follows: (a) the study employed a randomized controlled design; (b) only one SGARI was tested in each trial intervention; (c) mHSPC, mCRPC, and nmCRPC had all progressed during the study; (d) primary and secondary endpoints were included; and (e) either the hazard ratio (HR) or the number of events could be extracted from the text. The exclusion criteria were as follows: (a) publications were duplicated or contained poor-quality information; (b) the study contained insufficient primary data or incomplete study data; and (c) the publications were reviews, abstracts, commentaries, letters, or case reports.

### Data extraction and study quality

**Figure 2:**
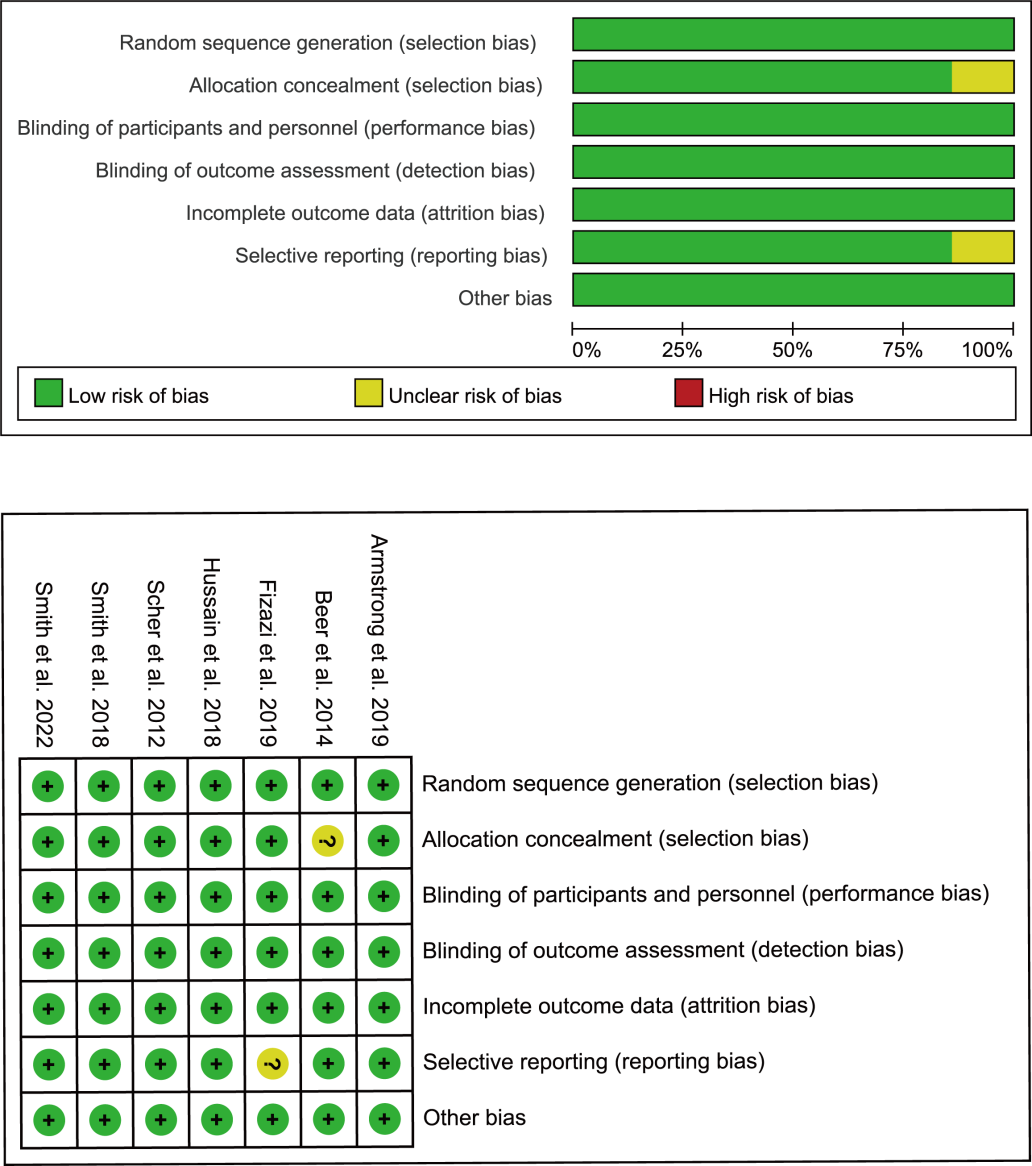
Bias risk assessment criteria for randomized controlled trials based on the Cochrane Collaborative Network.

First, two researchers independently screened literature and extracted data according to the established criteria. Reasons for excluding articles were also recorded. When disagreement arose, both parties negotiated or consulted with a third-party expert. Records included the first author, year of publication, clinical trial name, cancer characteristics, median age, interventions, median levels of prostate-specific antigen (PSA), and Eastern Cooperative Oncology Group. For each study, HR and confidence intervals (CI) were extracted for the reported primary and secondary endpoints, which included overall survival (OS), metastasis-free survival (MFS), rPFS, time to CRPC occurrence, time to pain progression, time to initiation of new antineoplastic therapy, time to PSA progression as defined by PCWG2 (16), and time to first skeletal-related event. We also extracted the number of overall adverse events (AEs) and noted the number of severe adverse events (grade ≥3). The quality of the included trials was assessed using the Cochrane Collaboration tool to assess the risk of bias in the randomized controlled trials (**Figure 2**) (17).

### Statistical analysis

Data were processed using Stata 16.0 and R 4.4.2 (18, 19). To investigate the effect of SGARI on selected primary and secondary endpoints, we first performed a direct meta-analysis. During model selection, a random effects model was chosen when I^2^ > 50%, and a fixed effects model was chosen when I^2^ < 50%. Next, a Bayesian network meta-analysis was performed using the GeMTC package to make indirect comparisons between different drugs (20). Rankograms were constructed to assess the ranking probability of each drug.

## Results

### Characteristics of included studies

During the initial selection process, 723 articles were included as candidates. After a stepwise screening process, seven articles were finally included in this meta-analysis (21–27). The article search and screening process is shown in **Figure 1**. The risk of bias evaluation for the final included studies is shown in **Figure 2**.** **A total of 9488 patients from all seven studies were assigned to receive either enzalutamide, apalutamide, darolutamide, or a placebo. The characteristics of the included studies are summarized in **Table 1**. Prostate cancer patients had been diagnosed by cytology and histology, and metastases were detected by bone scanning, contrast-enhanced computed tomography, or magnetic resonance imaging. **Table 2** summarizes the results for each study endpoint, for which an investigator graded the AEs according to the National Cancer Institute Common Terminology Criteria for Adverse Events. **Table 3** summarizes the results of indirect comparisons of SGARIs in each endpoint.

**Table 1:** Characteristics of the trails included in the meta-analysis.

First author	Year	Clinical trial	Cancer characteristics	Median age (yr)	Intervention arm	Control arm	Median PSA(ng/ml)	ECOG performance status score
Armstrong	2019	ARCHES	mHSPC	70	Enzalutamide plus ADT^a^	Placebo plus ADT	5.4	0 (78.0%) 1 (21.8%)
Smith	2022	ARASENS	mHSPC	67	Darolutamide plus ADT	Placebo plus ADT	30.3	0 (71.6%) 1 (28.4%)
Scher	2012	AFFIRM	mCRPC	NA	Enzalutamide plus ADT	Placebo plus ADT	NA	NA
Beer	2014	PREVAIL	mCRPC	NA	Enzalutamide plus ADT	Placebo plus ADT	NA	NA
Smith	2018	SPARTAN	nmCRPC	74	Apalutamide plus ADT	Placebo plus ADT	NA	NA
Hussain	2018	PROSPER	nmCRPC	74	Enzalutamide plus ADT	Placebo plus ADT	11.1	0 (80) 1 (20)
Fizazi	2019	ARAMIS	nmCRPC	74	Darolutamide plus ADT	Placebo plus ADT	9.0	0 (68%) 1 (32%)

NA, Not available.

a Androgen deprivation therapy (ADT): Surgical (bilateral orchiectomy) or chemical (pharmaceutical) interventions resulting in the reduction of serum testosterone or blockade of the androgen receptor.

**Table 2:** Summary results of the trial endpoints included in the analysis.

Adverse events≥3(n)	Adverse events(n)		Time to first skeletal-related event(mo)	Time to PSA progression (mo)	Time to initiation of new antineoplastic threapy (mo)	Time to pain progression (mo)	Time to castration-resistant prostate cancer (mo)	Radiographic Progression-free Survival (mo)	Metastasis free survival(mo)	Overall survival (mo)		
139	487	Enzalutamide(n=572)	_	_	30.2	8.3	NR	_	_	NR	Enzalutamide(n=574)	ARCHES
147	493	Placebo(n=574)	_	_	NR	8.3	13.8	_	_	NR	Placebo(n=576)	
			_	_	0.28 (0.20-0.40)	0.92 (0.78-1.07)	0.28 (0.22-0.36)	_	_	0.81 (0.53-1.25)	HR (95% CI)	
			_	_	<0.001	0.2715	<0.001	_	_	0.3361	P	
458	649	Darolutamide (n=652)^a^	_	_	NR	NR	NR	_	_	NE	Darolutamide (n=651)	ARASENS
439	643	Placebo (n=650)	_	_	25.3	27.5	19.1	_	_	48.9	Placebo (n=654)	
			_	_	0.39 (0.33-0.46)	0.79 (0.66-0.95)	0.36 (0.30-0.42)	_	_	0.68 (0.57-0.80)	HR (95% CI)	
			_	_	<0.001	0.01	<0.001	_	_	<0.001	P	
363	785	Enzalutamide(n=800)	16.7	8.3	_	_	_	8.3	_	–	Enzalutamide (n=800)	AFFIRM
212	390	Placebo (n=399)	13.3	3.0	_	_	_	2.9	_	–	Placebo (n=399)	
			0.69 (0.57-0.84)	0.25(0.20-0.30)	_	_	_	0.40 (0.35-0.47)	_	0.63 (0.53-0.75)	HR (95% CI)	
			<0.001	<0.001	_	_	_	<0.001	_	<0.001	P	
374	844	Enzalutamide (n=871)	31.1	11.2	_	_	_	_	_	–	Enzalutamide (n=872)	PREVAIL
313	787	Placebo (n=844)	31.3	2.8	_	_	_	_	_	–	Placebo (n=845)	
			0.72 (0.61-0.84)	0.17 (0.15-0.20)	_	_	_	0.19 (0.15-0.23)	_	0.71(0.60-0.84)	HR (95% CI)	
			<0.001	<0.001	_	_	_	<0.001	_	<0.001	P	
362	775	Apalutamide (n=803)	_	NR	_	_	_	_	40.5	NR	Apalutamide (n=806)	SPARTAN
136	371	Placebo (n=398)	_	3.7	_	_	_	_	16.2	39.0	Placebo (n=401)	
			_	0.06 (0.05-0.08)	_	_	_	_	0.28 (0.23-0.35)	0.70 (0.47-1.04)	HR (95% CI)	
			_	NR	_	_	_	_	<0.001	NR	P	
292	808	Enzalutamide (n=930)	_	37.2	_	_	_	_	36.6	NR	Enzalutamide (n=933)	PROSPER
109	360	Placebo (n=465)	_	3.9	_	_	_	_	14.7	NR	Placebo (n=468)	
			_	0.07 (0.05-0.08)	_	_	_	_	0.29 (0.24-0.35)	0.80 (0.58-1.09)	HR (95% CI)	
			_	<0.001	_	_	_	_	<0.001	–	P	
236	794	Darolutamide (n=954)	_	33.2	_	_	_	_	40.4	NR	Darolutamide (n=955)	ARAMIS
108	426	Placebo (n=554)	_	7.3	_	_	_	_	18.4	NR	Placebo (n=554)	
			_	0.13 (0.11-0.16)	_	_	_	_	0.41 (0.34-0.50)	0.71 (0.50-0.99)	HR (95% CI)	
			_	<0.001	_	_	_	_	<0.001	<0.001	P	

NR, Median value not reached; NE, Not estimable; HR, hazard ratio.

a Three patients who underwent randomization never received the assigned trial treatment; all three patients were in the placebo group. One patient who was assigned to the placebo group but received darolutamide was included in the darolutamide group of the safety analysis set. -, not mentioned in the trial.

**Table 3:** Indirect comparison results for each SGARI.

	ARCHES and ARASENS	AFFIRM and PREVAIL	SPARTAN, PROSPER and ARAMIS		
	Enzalutamide vs. Darolutamide[HR(95% CI)]	Enzalutamide(after chemotherapy) vs. Enzalutamide(before chemotherapy)[HR(95% CI)]	Apalutamide vs. Enzalutamide[HR(95% CI)]	Apalutamide vs. Darolutamide[HR(95% CI)]	Enzalutamide vs. Darolutamide[HR(95% CI)]
Overall survival(mo)	1.19 (0.75-1.89)	0.89(0.70-1.13)	0.88(0.53-1.45)	0.99(0.58-1.66)	1.13(0.71-1.79)
Metastasis free survival(mo)	_	_	0.97(0.73-1.28)	0.68(0.51-0.91)	0.71(0.54-0.93)
Radiographic Progression-free Survival(mo)	_	2.11(1.62-2.73)	_	_	_
Time to castration-resistant prostate cancer(mo)	0.78(0.58-1.05)	_	_	_	_
Time to pain progression(mo)	1.16(0.92-1.48)	_	_	_	_
Time to initiation of new antineoplastic threapy(mo)	0.72(0.49-1.05)	_	_	_	_
Time to PSA progression(mo)	_	1.47(1.15-1.89)	0.86(0.61-1.20)	0.46(0.34-0.62)	0.54(0.40-0.73)
Time to first skeletal-related event(mo)	_	0.96(0.75-1.23)	_	_	_
OR(95% CI)					
Adverse events(n)	0.40 (0.10,1.61)	0.53 (0.20,1.39)	1.04 (0.56,1.93)	1.35 (0.74,2.47)	1.30 (0.88,1.91)
Adverse events≥3(n)	0.82 (0.58,1.17)	0.57 (0.42,0.78)	1.06 (0.74,1.51)	1.17 (0.81,1.67)	1.10 (0.77,1.58)

HR, hazard ratio; OR, odds ratio. -, not mentioned in the trial.

### mHSPC

**Figure 3:**
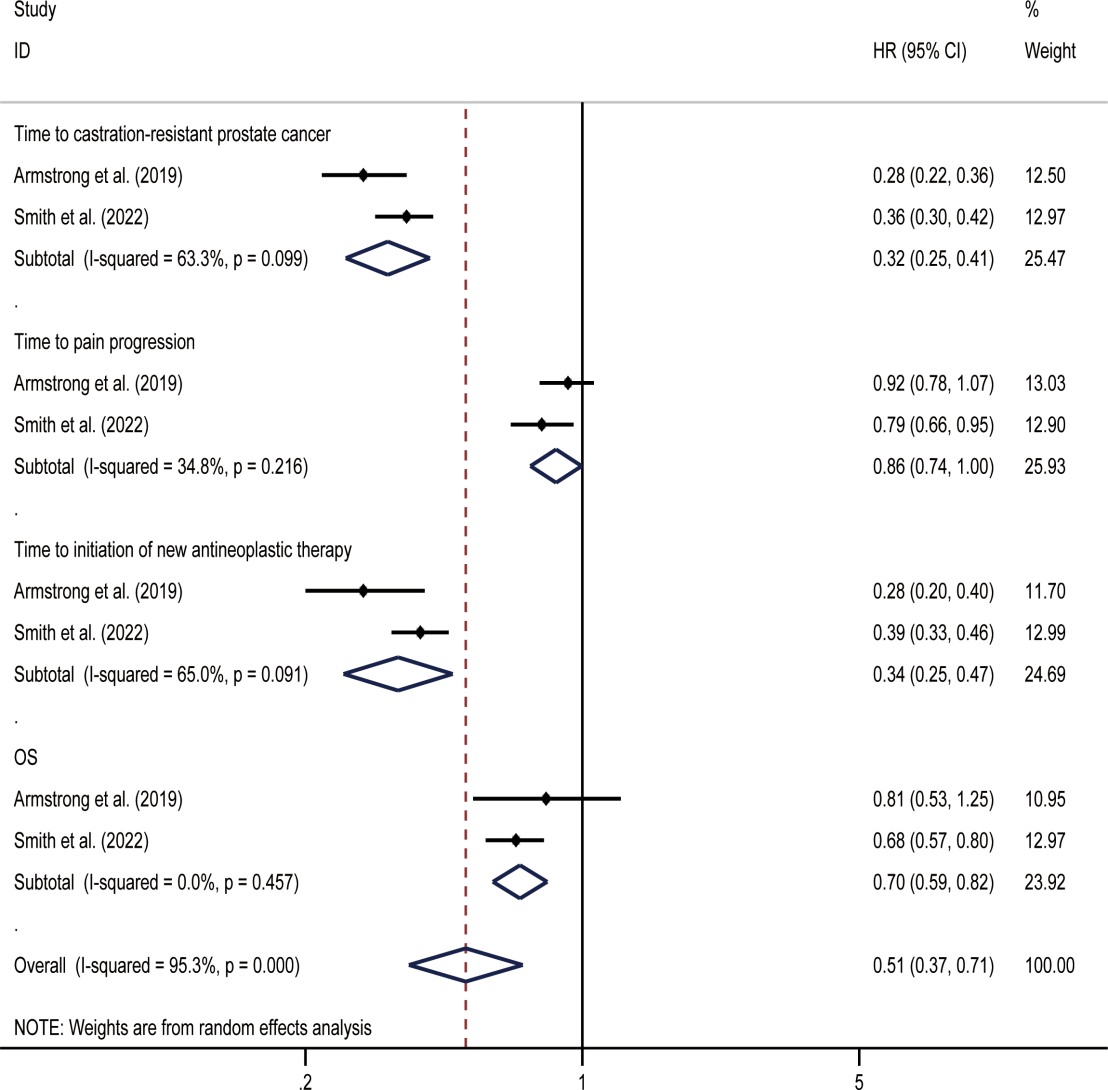
Results of a direct meta-analysis on the mHSPC study.

OS was the primary endpoint of both studies concerning mHSPC. In the original reported data, darolutamide was found to be beneficial in terms of OS (HR, 0.68; 95% CI, 0.57-0.80) and time to pain progression (HR, 0.79; 95% CI, 0.66-0.95) compared with the placebo. The random effect direct meta-analysis found that SGARI use was conducive to OS (HR, 0.70; 95% CI, 0.59-0.82) and improved the time to CRPC (HR, 0.32; 95% CI, 0.25-0.41), as well as the time to the initiation of new antineoplastic therapies (HR, 0.34; 95% CI, 0.25-0.47). However, SGARI did not improve the time to pain progression (HR, 0.86; 95% CI, 0.74-1.00) (**Figure 3**). In the indirect comparison, we did not find any differences between the efficacies of enzalutamide and darolutamide for these endpoints (**Table 3**). We calculated the odds ratio (OR) and CIs using the number of reported AEs to indirectly compare the utility of SGARIs based on AE incidences. Through indirect comparison, we found that the incidences of all occurring AEs and AEs of grade ≥ 3 did not significantly differ between enzalutamide and darolutamide.

**Figure 4:**
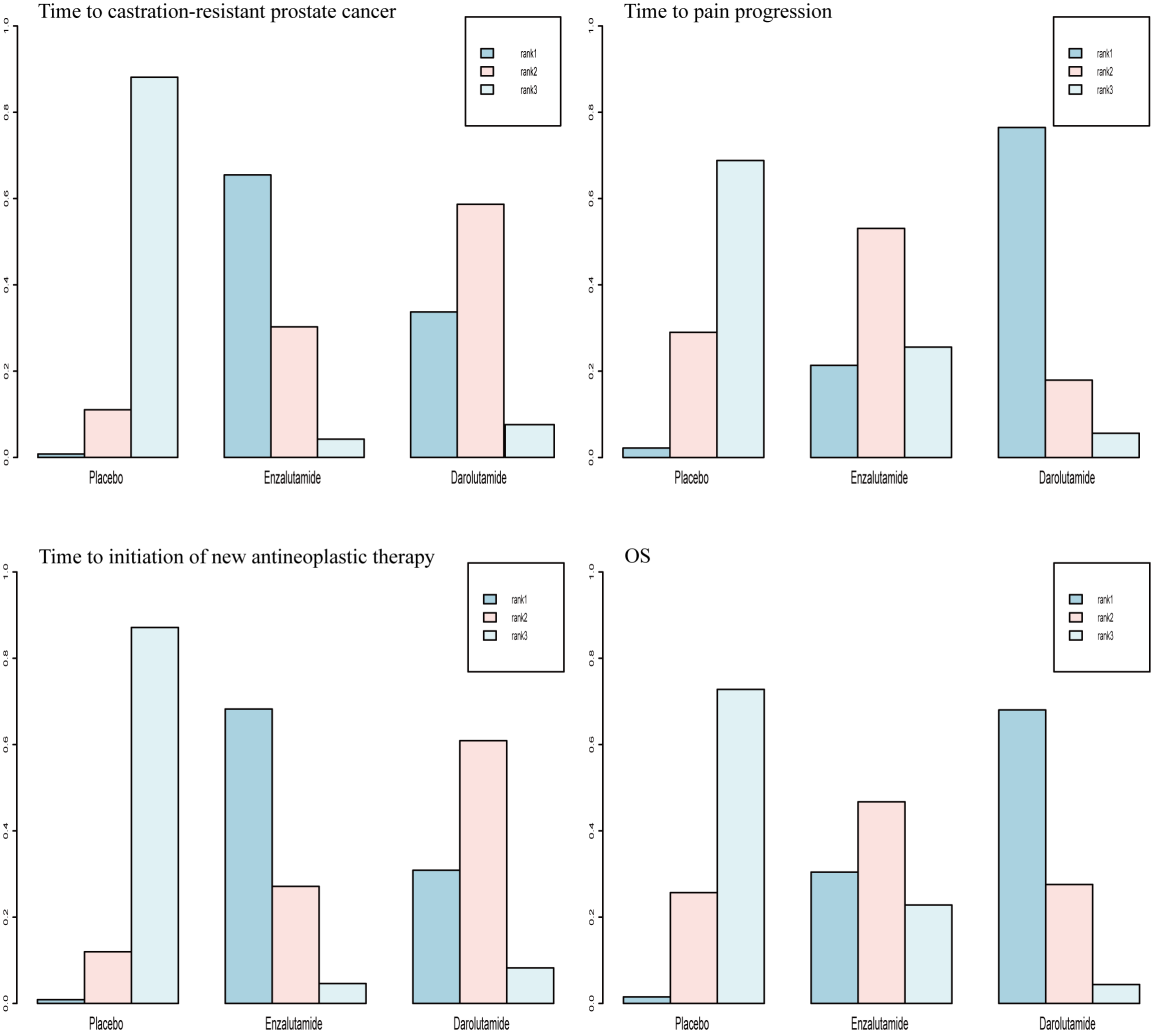
Rank probabilities of the SGARIs studied in the mHSPC study.

For OS and time to pain progression, darolutamide had a 68.0% and 76.5% probability, respectively, of being the preferred treatment option based on the rank probability analysis. For the time to CRPC occurrence and time to the initiation of new antineoplastic therapies, there was a 65.5% and 68.2% probability, respectively, that enzalutamide would be the preferred treatment option (**Figure 4**).

### nmCRPC

**Figure 5:**
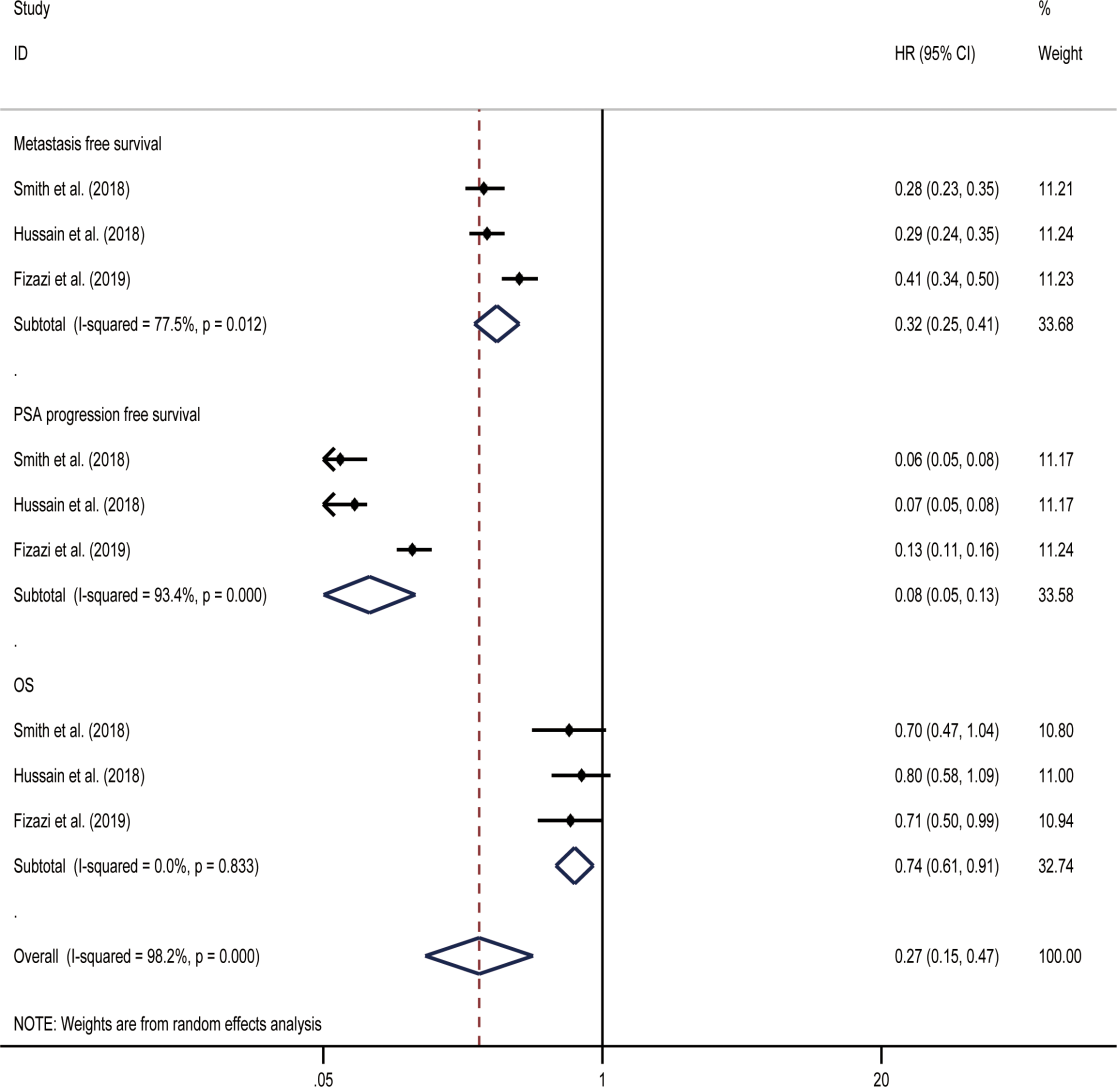
Results of a direct meta-analysis on the nmCRPC study.

MFS was the primary endpoint in all three studies concerning nmCRPC. The random effect direct meta-analysis showed that ARIs were beneficial in terms of MFS (HR, 0.32; 95% CI, 0.25-0.41), OS (HR, 0.08; 95% CI, 0.05-0.13), and time to PSA progression (HR, 0.74; 95% CI, 0.61-0.91) (**Figure 5**). When indirectly compared to darolutamide, both apalutamide and enzalutamide improved MFS and the time to PSA progression, with no significant differences in the extent of improvement (**Table 3**). In addition, indirect comparisons revealed that only darolutamide had a better OS than the placebo; apalutamide, enzalutamide, and darolutamide did not significantly differ in this regard. We also found no significant difference in the AEs overall or incidence of AEs of grade ≥ 3 for the three drugs.

**Figure 6:**
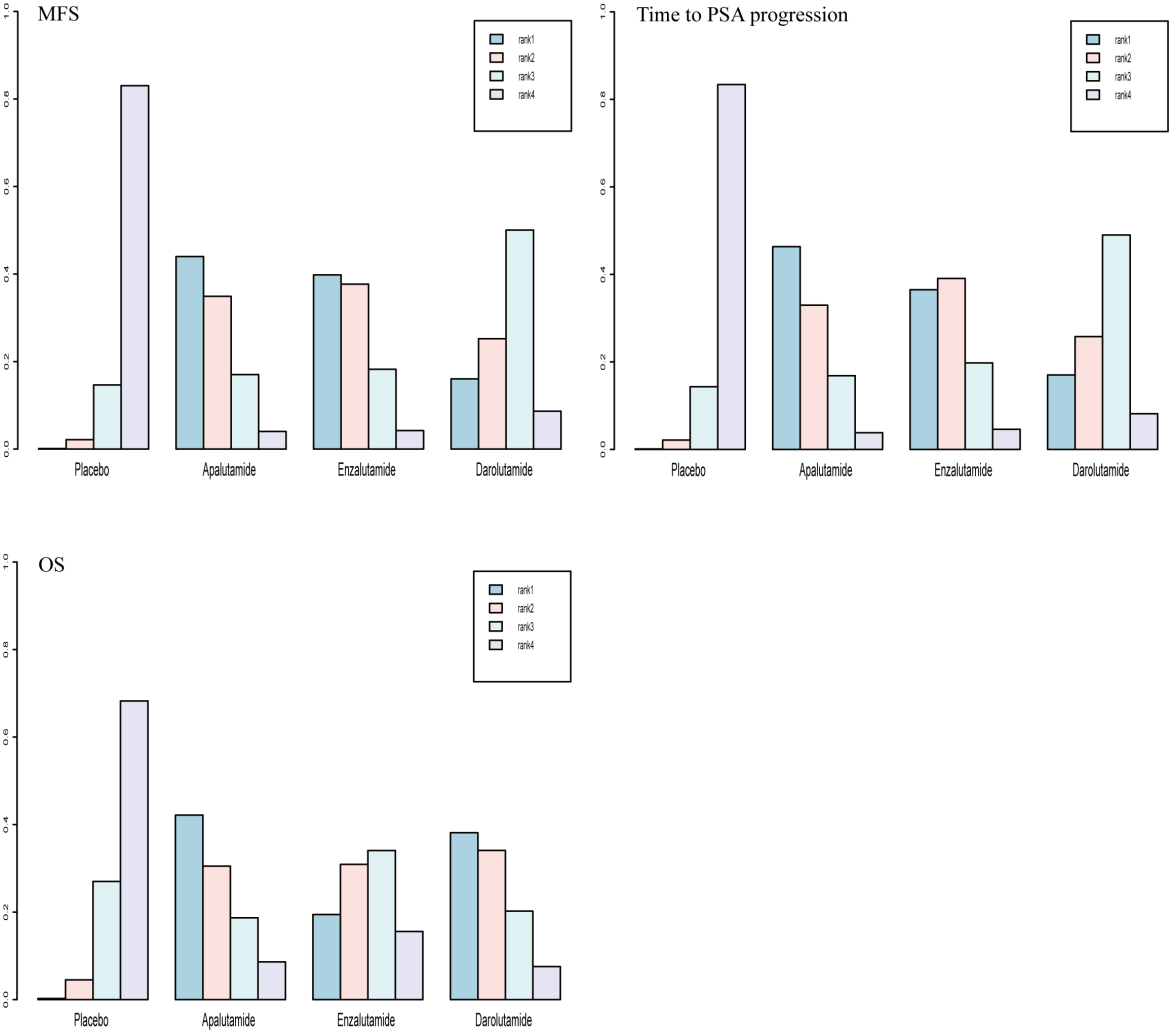
Rank probabilities of the SGARIs studied in the nmCRPC study.

Rank-probability analysis revealed that apalutamide had a 44.0% probability of being the preferred treatment option, followed by enzalutamide and darolutamide. For OS, apalutamide had a 42.2% probability of being the preferred treatment option, followed by darolutamide and enzalutamide. For time to PSA progression, apalutamide had a 46.3% probability of being the treatment of choice, followed by enzalutamide and darolutamide (**Figure 6**).

### mCRPC

**Figure 7:**
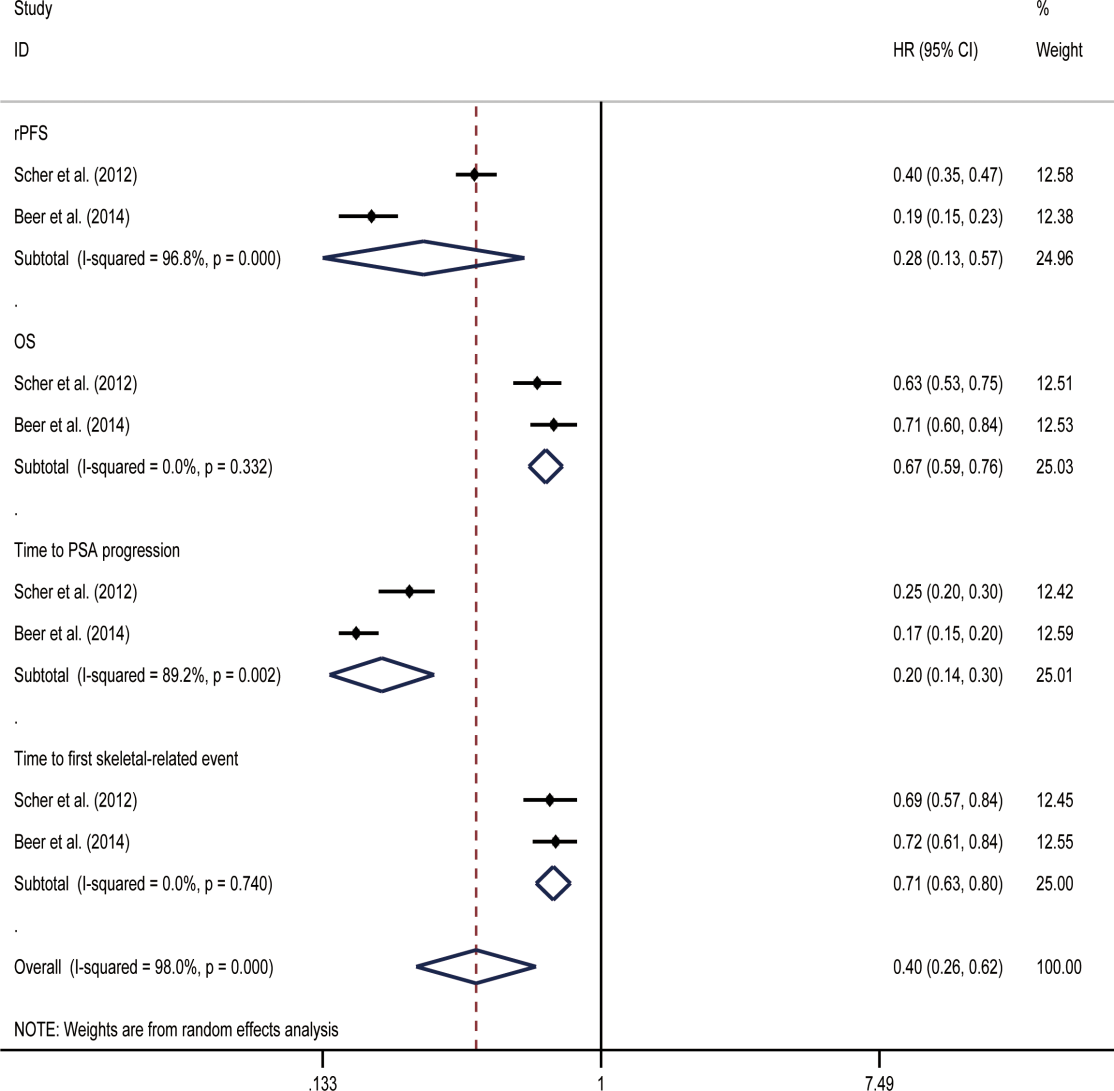
Results of a direct meta-analysis on the mCRPC study.

OS and rPFSs were the primary endpoints of the studies on mCRPC that were included in our analysis. Initial data from the included study showed that both before and after chemotherapy, enzalutamide was beneficial for all included endpoints when compared with placebos (**Table 2**). In random effect direct meta-analysis SGARI improved OS (HR, 0.67; 95% CI, 0.59-0.76), rPFS (HR, 0.28; 95% CI, 0.13-0.57), time to PSA progression (HR, 0.20; 95% CI, 0.14-0.30), and time to first skeletal-related event (HR, 0.71; 95% CI, 0.63-0.80) (**Figure 7**). The indirect comparison revealed no difference in OS improvement and the time to first skeletal-related event development when using enzalutamide before or after chemotherapy (**Table 3**). Use of enzalutamide before chemotherapy yielded more favorable rPFS and time to PSA progression than using enzalutamide after chemotherapy. There was no difference in the incidence of all AEs for using enzalutamide before or after chemotherapy. However, using enzalutamide after chemotherapy had improved the AEs (grade ≥ 3) relative to its use before chemotherapy.

**Figure 8:**
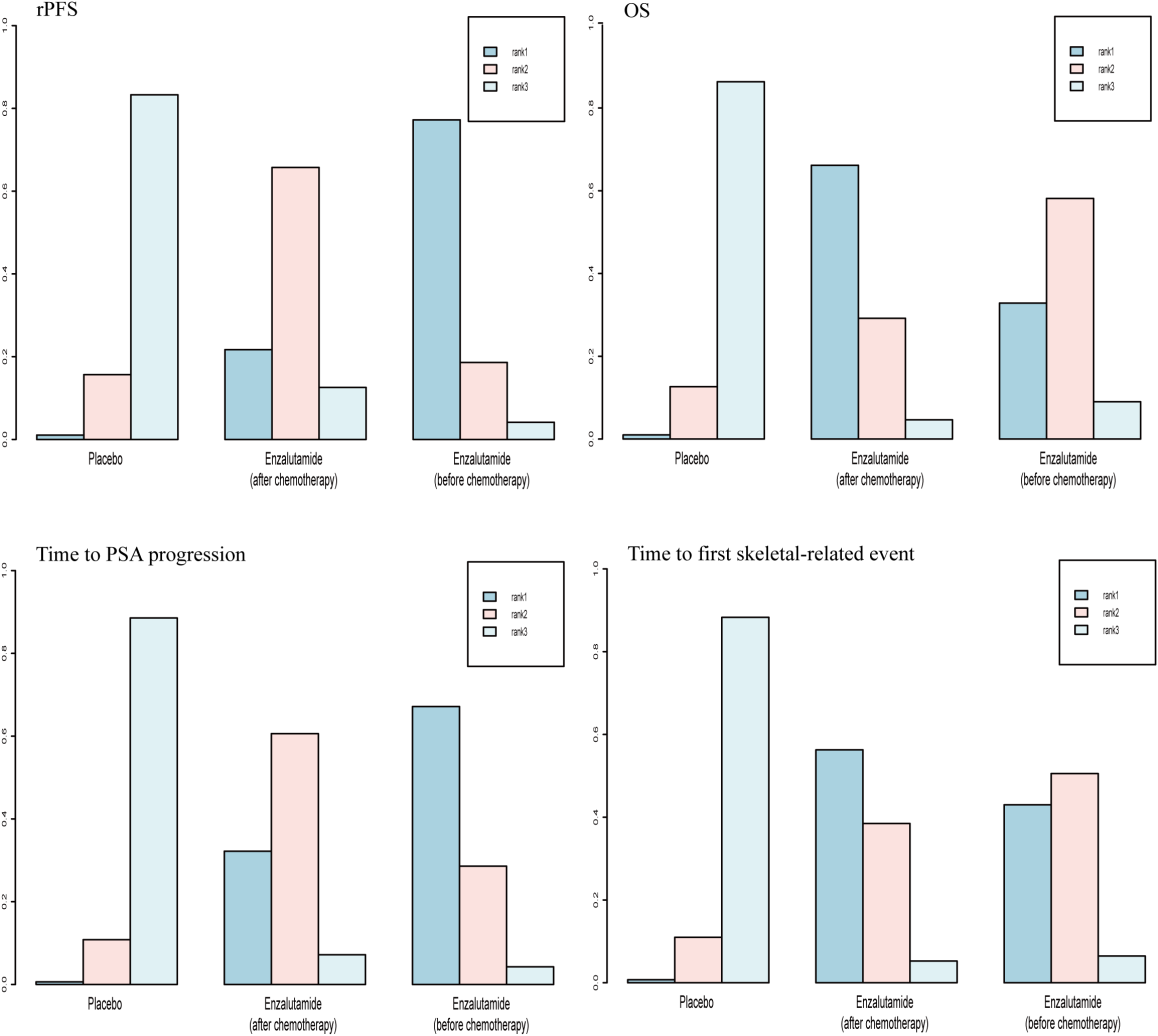
Rank probabilities of the SGARIs studied in the mCRPC study.

For OS and time to first skeletal-related event, there was a 66.2% and 56.3% probability, respectively, that enzalutamide would be the preferred regimen after chemotherapy. For rPFS and time to PSA progression, there was a 77.2% and 67.2% probability, respectively, of enzalutamide being the preferred regimen before chemotherapy (**Figure 8**).

## Discussion

According to the methodology of GLOBOCAN 2020 (28), a model estimates that, by 2022, over 120,000 of new patients with prostate cancer will be diagnosed in China and more than 210,000 will likewise be diagnosed in the United States (29). Three SGARIs have greatly affected prostate cancer treatment as tolerable and efficacious alternatives to chemotherapy since their approval by the FDA in 2013 (30). This meta-analysis selected the appropriate study endpoints to compare SGARI efficacy in mHSPC, mCRPC, and nmCRPC. We found that the SGARIs selected in three prostate cancer trials (enzalutamide and darolutamide for mHSPC; enzalutamide, apalutamide, and darolutamide for nmCRPC; enzalutamide for mCRPC) had prolonged OS for mHSPC and mCRPC, as well as MFS for nmCRPC. The results of the first direct meta-analysis showed that patients on SGARI had benefited significantly regarding all included study endpoints except the time to pain progression. In the mHSPC trial, in which OS was the primary endpoint, our network meta-analysis ranking showed no significant difference in efficacy between enzalutamide and darolutamide. In the mCRPC trial, OS and rPFS were selected as the primary endpoints in the AFFIRM and PREVAIL trials, respectively; therefore, we considered both endpoints as primary endpoints. There was no significant difference in indirect effects on OS between pre- and post-chemotherapy enzalutamide. Yet, pre-chemotherapy enzalutamide improved rPFS, making it the more favorable choice. For trials regarding nmCRPC, the OS analysis may not have been representative, and none of the three studies reached the median OS in the intervention group. In this case, MFS would have been clearly better suited as the primary endpoint, showing improved efficacy with apalutamide and enzalutamide compared to darolutamide. However, by indirect comparison, we were unable to obtain a difference in effectiveness between apalutamide and enzalutamide.

When SGARIs show similar efficacies, the secondary endpoints of the trial, patient quality of life, and the side effects of drug treatment should be considered. Regarding time to PSA progression as an important secondary endpoint, Saad et al. found that enzalutamide treatment reduced the risk of clinically meaningful metastasis or death in patients with nmCRPC and rapidly rising PSA levels, regardless of PSA progression status determined by the PCWG2 criteria (31). Stable PSA levels at radiographic progression may be associated with more aggressive, AR-independent variants of prostate cancer (32), and nearly a quarter of patients in the PREVAIL trial had stable PSA levels at the time of imaging progression. At the same time, radiographic progression often occurs without PCWG2-defined PSA progression, suggesting that any increase in PSA levels may warrant closer monitoring. The aforementioned observations further rationalize a prospective reassessment of PSA progression thresholds using different criteria. The most widely used questionnaires for assessing patient-reported outcomes are the Functional Assessment of Cancer Therapy-Prostate (FACT-P) questionnaire and the Brief Pain Inventory Short Form (BPI-SF). Using these questionnaires, the same drugs have been evaluated with varying results in different prostate cancers. In the AFFIRM trials, there was a significant difference in quality-of-life improvement, defined as a 10-point gain on two consecutive measurements at least three weeks apart. In the PREVAIL trials conducted in the pre-docetaxel setting, enzalutamide delayed the time to FACT-P score degradation, defined as a 10-point decrease from the baseline score. However, in the ARCHES and PROSPER trials, neither the FACT-P total score nor the median time to score degradation improved significantly after enzalutamide treatment. As assessed by the BPI-SF scale, the AFFIRM and PREVAIL trials reported fewer patients with pain progression in the enzalutamide group than in the control group.

Systemic prostate cancer therapy may increase the risk of AEs. For patients, the appropriate selection of SGARI therapies can possibly mitigate adverse event risks. Fatigue was the most common AE in all SGARI trials, and cardiac disorders were the greatest concern among clinically significant AEs. A meta-analysis showed that treatment with ARIs was associated with a significant increase in cardiovascular toxicity (33). In line with the current view that cardiovascular risk may differ between the newer ARIs, studies have recommended to monitor for cardiovascular risk factors and cardiovascular events in patients taking apalutamide and enzalutamide, though this may be unnecessary in patients taking darolutamide (34). In our study, however, we found no differences in the efficacies of the three SGARIs regarding adverse events. Additionally, an ongoing multicenter longitudinal Cog-Pro study aims to assess the incidence of cognitive impairment in older men treated with anti-androgenic drugs. This study could help improve the care of older patients with prostate cancer who experience AEs (35).

The findings of this review should be interpreted within the context of its limitations. First, both apalutamide and darolutamide have only one trial, and many of the conclusions rely on indirect comparisons, which cannot substitute for direct comparisons; therefore, these results should be interpreted cautiously. Additionally, for indirect comparisons, we used HR and OR; whether different utility measures can provide interpretations of drug efficacy requires further validation. Second, we did not perform a subgroup analysis by race and ethnicity. Due to the lack of raw data, we could not determine whether race, a high-risk factor for prostate cancer, impacted the results. Additionally, the mechanisms behind AEs in prostate cancer remain unclear. We only performed indirect comparisons of AEs overall and AEs with grades ≥3; each AE was not analyzed in detail. Therefore, we look forward to data from larger samples to help understand the risk of drug-drug interactions in ARIs as part of optimal patient management (36).

In conclusion, our study had carefully screened evidence from high-quality randomized controlled trials to demonstrate the utility of SGARI in prolonging survival in patients with high-risk prostate cancer. We further provided a basis for clinicians to improve specific endpoints. In the future, we also expect to investigate whether combinations of SGARI and other drugs can be more effective than SGARI alone for the treatment of high-risk prostate cancer.

## Conclusion

According to our findings, the SGARIs had prolonged OS for mHSPC, OS and rPFS for mCRPC, as well as MFS for nmCRPC. First, there was no significant difference in OS improvement between patients with mHSPC treated with enzalutamide and darolutamide. In addition, enzalutamide and apalutamide were the most effective treatment options for improving MFS in patients with nmCRPC. Further, both pre- and post-chemotherapy enzalutamide use improved OS in mCRPC patients, but for improving rPFS pre-chemotherapy use of enzalutamide should be preferred. In mHSPC and nmCRPC, the AE profile of the 3 SGARIs were not significantly different. However, in the face of AEs of grade ≥ 3 in mCRPC, the best option was to use enzalutamide after chemotherapy.

## Author contributions

XC and QW designed the study and analyzed the data. YP, YL, and HZhang revised the images. SW, MX and HZhou performed the literature search and collected data for the manuscript. XL revised the manuscript. All authors contributed to the article and approved the submitted version.
